# Ensiling Improved the Colonization and Degradation Ability of *Irpex lacteus* in Wheat Straw

**DOI:** 10.3390/ijerph192013668

**Published:** 2022-10-21

**Authors:** Dongze Niu, Peng Zhu, Tingting Pan, Changyong Yu, Chunyu Li, Jianjun Ren, Chuncheng Xu

**Affiliations:** 1 Changzhou Key Laboratory of Biomass Green, Safe & High Value Utilization, National-Local Joint Engineering Research Center of Biomass Refining and High-Quality Utilization, Institute of Urban and Rural Mining, Changzhou University, Changzhou 213164, China; 2 College of Engineering, China Agricultural University, Beijing 100083, China; 3 School of Petrochemical Engineering, Changzhou University, Changzhou 213164, China; 4 Shandong Institute of Standardization, Jinan 250014, China

**Keywords:** ensiling, wheat straw, white rot fungi, enzymic hydrolysis, lignin

## Abstract

To develop a non-thermal method to replace steam autoclaving for white-rot fungi fermentation, *Irpex lacteus* spawn was inoculated in wheat straw (WSI) or ensiled WS (WSI) at varying ratios of 10%, 20%, 30%, 40%, and 50%, and incubated at 28 °C for 28 days to determine the effects of the ensiling and inoculation ratio on the colonization and degradation ability of *Irpex lacteus* in wheat straw (WS). The results demonstrate that ensiling effectively inhibited the growth of aerobic bacteria and molds, as well as other harmful microorganisms in WS, which created a favorable condition for the growth of *I. lacteus*. After the treatment of *I. lacteus*, the pH of EWSI decreased to below 5, while that of WSI, except for the feedstocks of WSI-50%, was around 7, indicating that *I. lacteus* colonized well in the ensiled WS because the substrates dominated by *I. lacteus* are generally acidic. Correspondingly, except for the molds in WSI-50% samples, the counts of other microorganisms in WSI, such as aerobic bacteria and molds, were significantly higher than those in EWSI (*p* < 0.05), indicating that contaminant microorganisms had a competitive advantage in non-ensiled substrates. Incubation with *I. lacteus* did not significantly affect the cellulose content of all samples. However, the NDS content of EWSI was significantly higher than that of WSI (*p* < 0.05), and the hemicellulose and lignin contents were significantly lower than the latter (*p* < 0.05), except for the NDS and hemicellulose contents of WSI-50% samples. Correlation analysis revealed a stronger negative correlation between NDS content and the contents of hemicellulose, cellulose, and lignin in EWSI, which could be caused by the destruction of lignin and hemicellulose and the conversion from structural carbohydrates to fungal polysaccharides or other compounds in NDS form. Even for WSI-50% samples, the sugar yield of WS treated with *I. lacteus* improved with an increasing inoculation ratio, but the ratio was not higher than that of the raw material. However, the sugar yield of EWSI increased by 51–80%, primarily owing to the degradation of lignin and hemicellulose. Above all, ensiling improves the colonization ability of *I. lacteus* in WS, which promotes the degradation of lignin and hemicellulose and the enzymic hydrolysis of cellulose, so combining ensiling and *I. lacteus* fermentation has promising potential in the pretreatment of WS.

## 1. Introduction

Wheat straw (WS) is an important renewable resource that can produce valuable products or raw materials, including ruminant feed, biomass fuel, and platform chemicals [1,2]. However, degradable nutrients in the stalks, such as fat, starch, and protein, are transferred to the seeds during the ripening process of wheat plants; hence, mature WS is mainly composed of cellulose, hemicellulose, and lignin with low nutritional value [3,4]. Lignin forms a physical barrier through intramolecular processes and cross-linking with hemicellulose molecules, which limits the utilization of sugar in straw by microorganisms and their secreted enzymes [3,5]. To improve the utilization efficiency of lignocellulose, many researchers are constantly attempting to change the physical and chemical structure of cell walls through physical, chemical, biological, and combined methods to break the enzymatic hydrolysis resistance barrier of lignocellulose and improve the application value of straw in feed, energy, and other fields [3]. Compared to physical and chemical methods, a biological method represented by white-rot fungi (WRF) fermentation has attracted wide attention because of its mild fermentation conditions and no by-product production [6,7].

WRF is a type of filamentous fungi that causes the white rot of wood due to their better ability to degrade lignin than that of cellulose, so WRF has potential applications in the pretreatment of lignocellulose [6,7,8]. WRF, such as *Irpex lacteus*, *Lentinus edodes,* and *Ceriporiopsis subvermispora*, is effective in selectively degrading lignin [2,9]. However, most studies were conducted under sterilization conditions, resulting in high pretreatment costs because of the requirements for autoclaving and aseptic conditions [7,10,11]. To simplify the fermentation process and reduce the production cost, researchers have attempted to increase the inoculation amount [12] and sterilize substrates using a CaO solution and pasteurization [13,14] and have proven their effectiveness in improving the colonization ability of WRF. However, the methods still require washing with a large quantity of water [14], a higher than 30% inoculation amount [12], or a relatively high temperature [13]. Therefore, developing economical, environmentally friendly, and efficient pretreatment methods is crucial to inhibit the growth of harmful microorganisms and improve the colonization and degradation ability of WRF.

Ensiling is a technology that uses the anaerobic fermentation of lactic acid bacteria (LAB) to preserve high-water-content feed crops [15,16]. This technology not only effectively avoids loss caused by poor weather conditions during drying but also reduces the risk of fire during storage [17]. Studies have demonstrated that ensiling can increase the hydrophilicity of lignin and the number of small holes in the cell wall, thus improving the enzymatic digestibility of Napier grass and *Arundo donax* [18,19]. Although the anaerobic process has little effect on lignin content and cannot remove lignin’s blocking effect on cellulase [20], the pH and the growth of harmful microorganisms in ensiled substrates decreases significantly after several weeks of anaerobic fermentation, allowing most silages to retain an adequate quality after several days or even weeks of opening [21,22]. Our previous studies have confirmed that *I. lacteus* has strong resistance to foreign bacteria and acidic conditions, so it colonizes well in ensiled WS (EWS), corn stalk, and oat straw [23,24,25]. However, the effects of ensiling on inoculation ratio, chemical composition, and enzymic efficiency are still unclear.

Generally, fungus-colonized substrate is a cheap and easily available inoculum, so *I. lacteus* colonized in WS was inoculated into untreated WS or EWS at various proportions and incubated at 28 °C for 28 days in this study. Culturable microbes, chemical composition, and enzymatic hydrolysis of WS were measured to determine the benefits of ensiling and the optimal inoculation ratio for WRF fermentation, which will provide theoretical guidance and methodological reference for the large-scale application of *I. lacteus* fermentation in WS treatment.

## 2. Materials and Methods

### 2.1. Fungal Strain and Preparation of I. lacteus Spawn

The fungal strain (*I. lacteus* CGMCC 5.809) used in this experiment was obtained from China General Microbiological Culture Collection Center (CGMCC) in Beijing, China. Before the experiment, the strain was inoculated into a culture dish containing potato dextrose agar (PDA) medium and cultured at 28 °C for five days [2]. After the mycelium had colonized the culture dish, it was stored at 4 °C for further use.

The primary medium was comprised of 55% water, 39.5% wheat grains, 3% crushed WS, and 2.5% calcium sulfate. After sterilization at 121 °C for 20 min, ten 5 mm PDA plugs covered with *I. lacteus* mycelium were inoculated in 100 g primary medium and cultured at 28 °C for 5–7 days to make *I. lacteus* spawn [23]. The primary *I. lacteus* spawn (4%) was then inoculated in sterilized WS (121 °C, 20 min) with 70% water content and incubated at 28 °C for 28 days to obtain the secondary *I. lacteus* spawn, which were used as inoculants for subsequent fermentation.

### 2.2. Substrates and Ensiling Procedure

WS used in this experiment was obtained from the National Experiment Station for Precision Agriculture (40.22° N, 116.20° E, Beijing, China). The harvested WS was air-dried in the field before being collected, chopped to 1–2 cm with a hay cutter, and stored in a cool, dry place for subsequent ensiling or fermentation.

EWS was prepared from chopped WS according to a previous report [23]. Briefly, the moisture content of WS was adjusted to 55% and then the substrates were separated into polyethylene vacuum packaging bags, which were sealed with a vacuum packaging machine (Ouxin Packaging Machinery Co., Ltd., Zhejiang, China). All samples were stored in the dark at room temperature. After ensiling for 28 days, the samples were opened and sampled for microbial and chemical analysis and the remaining samples were used for subsequent fermentation with *I. lacteus*.

### 2.3. Fermentation with I. lacteus

The water content of WS and EWS was adjusted to 70% using tap water, and then they were separated into polyethylene fresh-keeping bags (Miaojie, Wuxi, Jiangsu, China). After that, 10%, 20%, 30%, 40%, and 50% of secondary *I. lacteus* spawn (*w*/*w*) were inoculated into WS- and EWS-containing bags. After uniform mixing, they were incubated at 28 °C for 28 days. The 50% inoculated groups were sampled at 7, 14, and 28 days, whereas the samples in other groups were only sampled at 28 days. Each treatment had three replicates.

### 2.4. Microbial Analysis

The analysis of culturable microorganisms was conducted according to previous reports [16,23]. Briefly, 10 g of raw materials or fermentation samples were put into an extraction bag with 90 mL of sterilized distilled water. After thoroughly shaking, 1 mL of the extracted solution was diluted and spread in nutrient agar medium (Nissui, Japan), de Man, Rogosa, and Sharpe (MRS) agar (Difco Laboratories, Detroit, MI, USA), and Rose Bengal medium (Aobox, Beijing, China) for counting aerobic bacteria, LAB, and fungi (molds and yeasts), respectively [23]. A Mettler Toledo S20K pH meter (Greifensee, Switzerland) was used to determine the pH of fermentation samples. To determine organic acid content, the remaining extracted solutions were filtered and stored at −20 °C.

### 2.5. Chemical Analyses and Enzymatic Hydrolysis

About 150 g of raw materials or fermented WS were removed from each treatment. After accurate weighing, they were dried in a drying oven at 65 °C for 48 h to determine dry matter (DM) content. The dried samples were shattered using a high-speed grinder with a 1 mm screen. The water-soluble carbohydrate (WSC) content was determined using the anthrone method [21]. Neutral detergent solute (NDS), neutral detergent fiber (NDF), acid detergent fiber (ADF), and acid detergent lignin (ADL) contents were analyzed using an Ankom 2000 automated fiber analyzer (Ankom Technology, Fairport, NY, USA) [2]. Hemicellulose and cellulose contents were calculated using the difference between NDF, ADF, and ADL. The sugar yield of raw and treated WS was determined using the revised method of the National Renewable Energy Laboratory (NREL) [23].

### 2.6. Statistical Analyses

All data were analyzed using one-way analysis of variance (ANOVA) in IBM SPSS Statistics 21.0 (IBM SPSS Inc., Chicago, IL, USA). Duncan’s multiple range test was used to compare different treatments and significance was declared when *p* < 0.05. The association between the parameters was evaluated using Spearman’s correlation analysis.

## 3. Results and Discussion

### 3.1. Chemical and Microbial Compositions of Raw Materials before Inoculation

Table 1 displays the chemical and microbial compositions of WS and EWS before inoculating *I. lacteus* spawn. The WSC content of raw WS was 5.15% DM and significantly decreased (*p* < 0.05) after ensiling for 28 days, which is mainly attributed to the conversion of WSC to organic acids by LAB. During the fermentation process, LAB counts increased from 3.52 to 6.87 log_10_ CFU/g FM, but molds decreased to a non-detectable level. The changes in chemical and microbial composition after ensiling are consistent with previous reports [15,23]. The growth of harmful microorganisms on the surface of WS was inhibited by the organic acid produced by LAB [26], indicating that ensiling is an effective method to reduce the abundance of harmful microorganisms in WS.

### 3.2. pH and Microbial Composition during Incubation with I. lacteus

**Figure 1:**
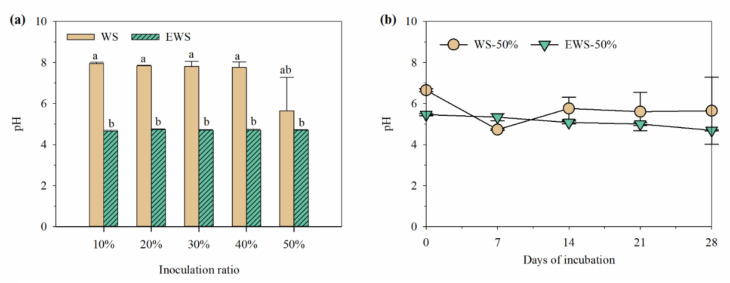
Effects of different treatment and fermentation days on the pH of wheat straw treated with *I. lacteus.* (**a**) treatment with different inoculating ratio; (**b**) treatment with 50% inoculating ratio at different days; mean ± SD (*n* = 3); values with different small letters differed (*p* < 0.05) among treatment.

Figure 1 depicts the pH and culturable microorganisms in different treatments. The inoculation amount had no significant effect on the pH of EWSI after 28 days of incubation (*p* > 0.05), which ranged around 4.7 (Figure 1a). For the control treatment, the decreased pH was only observed in substrates with 50% inoculation (*p* < 0.05). Unlike other aerobic microorganisms, WRF can produce organic acids and some other active chemicals to inhibit most coexisting microorganisms, so the pH of substrates is an important index to evaluate WRF growth [23,27]. In this study, pH variations reflect that ensiling promotes the proliferation of *I. lacteus* in WS, consistent with previous reports [23,24].

In treatments with 50% inoculum, the pH of WSI-50% was significantly higher than that of EWSI-50% (*p* < 0.05), but the pH of both groups reached a similar value with an extending fermentation time, indicating that *I. lacteus* can secrete organic acids and reduce the pH of substrates [28]. To summarize, ensiling can enhance the colonization ability of *I. lacteus* in WS and thus reduce the requirement of the inoculation amount, which is an effective strategy for providing a competitive advantage for WRF growth.

**Figure 2:**
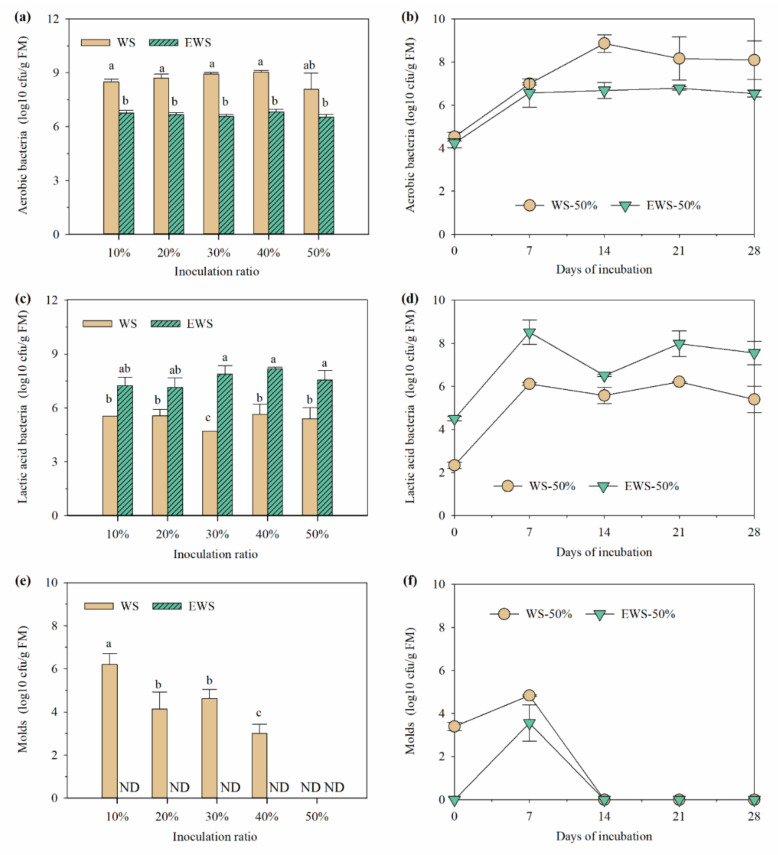
Effects of different inoculating ratio (**a**,**c**,**e**) and fermentation days (**b**,**d**,**f**) on the culturable microbes of wheat straw treated with *I. lacteus*.

Figure 2 displays the microorganism counts in various treatments. Similar to pH, ensiling significantly changed the microbial composition of WS treated with *I. lacteus*. After 28 days of fermentation, the counts of aerobic bacteria and molds in WSI were significantly higher than that in EWSI (*p* < 0.05), while the counts of LAB in WSI were significantly lower (*p* < 0.05). The aforementioned results are consistent with the change in microbial counts in WS before and after ensiling, reflecting that the change of microbial counts caused by ensiling in straw is irreversible.

In treatments with 50% inoculum, aerobic bacteria, LAB, and molds in WSI-50% and EWSI-50% significantly increased in the early fermentation stage (*p* < 0.05) but remained unchanged or significantly decreased in the middle and late stages. After 14 days of fermentation, mold count decreased below the detection limit. Throughout fermentation, the LAB count in EWSI-50% was significantly higher than that in WSI-50% (*p* < 0.05) and the counts of aerobic bacteria and mold were significantly lower than that in the latter (*p* < 0.05). WRF is known to coexist with some bacteria, thus promoting lignin degradation [29]. Although there is no evidence of symbiosis between WRF and LAB, straw fermented with WRF can be effectively ensiled [27,30]. Therefore, the metabolites of WRF do not inhibit LAB. On the contrary, some molds have an antagonistic relationship with WRF [31], so their lower counts in EWSI indicate that *I. lacteus* can continue the inhibitory effect on mold caused by ensiling.

### 3.3. Chemical Composition during Incubation with I. lacteus

**Figure 3:**
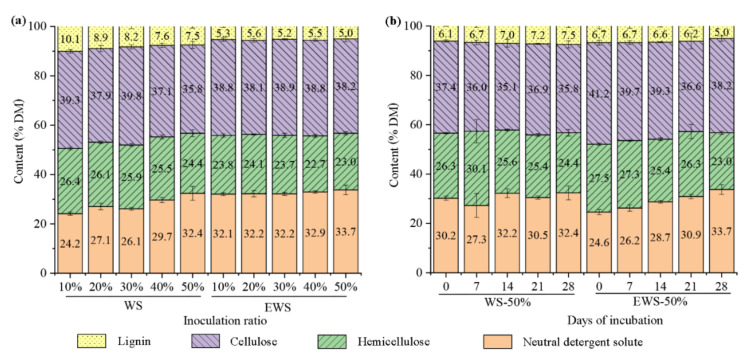
Effects of different treatment (**a**) and fermentation days (**b**) on the chemical composition of wheat straw treated with *I. lacteus*.

Figure 3 depicts the chemical composition of WS in various treatments. In the samples with different inoculation, the NDS content in WSI increased with an increasing inoculation amount and the value of WSI-50% reached 32.4%, which is 14% higher than that of untreated WS and had no significant difference compared to that of EWS. NDS refers to components that can be washed with a neutral detergent solution, including starch, soluble sugar, pectin, protein, soluble phenols, lipids, and ash [2]. Because NDS components are mostly degradable, *I. lacteus* fermentation significantly improved the nutritional value of WS in WSI-50% and all EWSI samples. Contrary to NDS content, the hemicellulose content of WSI-50% and EWSI samples was significantly lower than that of other samples (*p* < 0.05) because WRF has a strong ability to degrade hemicellulose [32]. There was no significant difference in the cellulose and lignin contents of EWSI with different inoculation amounts. Notably, the cellulose content of WSI-50% was significantly lower than that of EWSI but the lignin content of WSI-50% was higher. The co-culture of some microorganisms with WRF is reported to increase the activities of cellulase, xylose, and chitinase [8]. Combined with the higher counts of other microorganisms, we inferred that their existence promoted the degradation of cellulose and other nutrients, increasing the relative content of lignin.

In the treatments with 50% inoculum, the contents of cellulose and hemicellulose revealed a downward trend but the contents of NDS had an upward trend. Compared to day 0, the NDS content of EWSI-50% at day 28 increased by 37%. However, the changes in lignin content in WSI-50% and EWSI-50% had an opposite trend. After 28 days of fermentation, the lignin content of WSI-50% increased by 23.0%, while that of EWSI-50% decreased by 25.4%. The findings indicate that, although a higher inoculation amount is an effective method to promote the growth and degradation of WRF [12], the presence of other microorganisms promotes the degradation of cellulose, hemicellulose, and other nutrients, resulting in higher dry matter loss and thus limiting the decrease of lignin content. Ensiling before fermentation with *I. lacteus* can effectively inhibit the consumption of nutrients by other microorganisms.

To clarify the relationships among different chemical compositions during fermentation with *I. lacteus*, their correlations in WSI and EWSI were analyzed (Table 2). In both groups, the NDS content was negatively correlated with hemicellulose, cellulose, and lignin content, reflecting that a part of the degraded hemicellulose, cellulose, and lignin was converted into NDS, which is easier to digest and utilize, rather than being completely consumed by the microorganisms. The correlation between hemicellulose and NDS in EWSI was stronger (*R* = 0.928, *p* < 0.01) than that of WS. Combined with the low hemicellulose contents in EWS, *I. lacteus* fermentation is demonstrated to efficiently convert hemicellulose to NDS in EWS. In addition, a strong positive correlation was observed between the contents of cellulose and lignin in WSI (*R* = 0.642, *p* < 0.05). In contrast, a strong positive correlation was observed between hemicellulose and lignin in EWSI (*R* = 0.920, *p* < 0.01). In lignocellulose, cellulose and lignin are the most difficult to degrade [5]. Given the low NDS content in WS, the high correlation between cellulose and lignin is thought to be mainly caused by an increase in their relative content. In contrast, the loss of NDS and other substances in EWSI was less, and hemicellulose and lignin formed a spatial network structure in straw [3,7]. Therefore, the degradation of hemicellulose or lignin by WRF destroyed the network structure, resulting in their simultaneous decrease, which is the primary reason for the strong correlation between hemicellulose and lignin in EWS.

### 3.4. Sugar Yield of Enzymatic Hydrolysis before and after Incubation with I. lacteus

**Figure 4:**
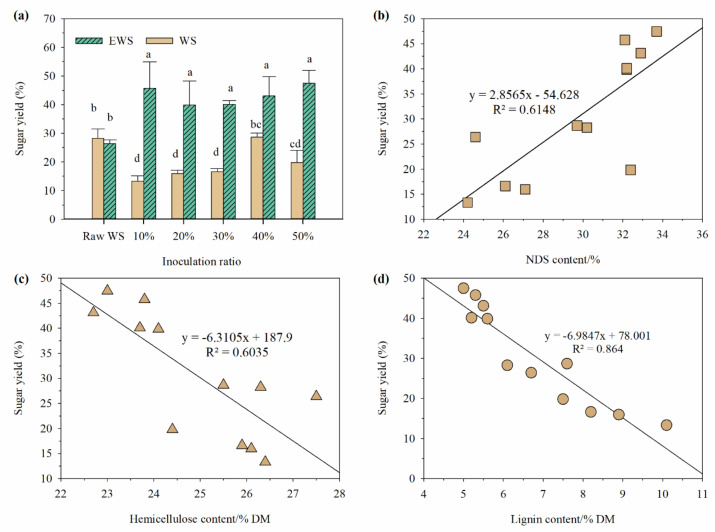
Effects of inoculating ratio on the enzymatic digestibility of wheat straw treated with *I. lacteus* (**a**) and its correlation with main components (**b**,**c**,**d**).

Figure 4 depicts the sugar yields of various raw materials and treatments. The sugar yield of WS after 72 h of enzymatic hydrolysis was 28% and ensiling had no significant effect on the value (*p* > 0.05). After fermentation with *I. lacteus* for 28 days, the sugar yield of samples in WSI below 40% inoculation significantly decreased (*p* < 0.05) and that of WSI-50% had no significant difference with raw materials (*p* > 0.05). In contrast, the sugar yield of samples in EWSI increased by 40% to 47% (*p* < 0.05), which is equivalent to the sugar yield of WS delignified with 1.5% NaOH or 80% p-toluenesulfonic acid [33]. Further analysis reveals that sugar yield was positively correlated with NDS content (*p* < 0.01) and negatively correlated with hemicellulose and lignin contents (*p* < 0.01) but no significant correlation was observed between cellulose content and sugar yield. Considering the negative correlation between lignin and hemicellulose contents and the positive correlation between lignin and NDS contents, we inferred that the increased sugar yield of WS after fermentation with *I. lacteus* was mainly caused by the degradation of hemicellulose and lignin [2,10]. Above all, *I. lacteus* can effectively colonize in EWS and destroy the complex network of cellulose and hemicellulose, promoting the enzymatic hydrolysis of cellulose, so combining ensiling with *I. lacteus* fermentation is an effective method for high-value uses of WS.

## 4. Conclusions

Ensiling significantly altered the microbial community structure on the surface of WS, inhibited the growth of harmful microorganisms, and created favorable conditions for subsequent fermentation. Although *I. lacteus* can colonize in WS without ensiling when the inoculation amount is greater than 50%, the abundance of harmful microorganisms, such as aerobic bacteria, was high, making it impossible to improve the lignin degradation rate and sugar yield of WS. However, even at 10% inoculation, *I. lacteus* colonized well in EWS, and the lignin degradation rate and enzymatic hydrolysis sugar yield significantly improved. Therefore, inoculating 10% *I. lacteus* colonized in WS is an effective, economical, readily available inoculant for EWS.

## Figures and Tables

**Table 1:** Chemical and microbial compositions of the intact and ensiled wheat straw before inoculating *I. lacteus* spawn.

Item	Wheat Straw	Ensiled Wheat Straw
WSC (% DM)	5.15 ± 0.243	0.59 ± 0.038
NDS (% DM)	28.4 ± 0.23	25.2 ± 0.36
Hemicellulose (% DM)	27.9 ± 0.28	29.7 ± 0.31
Cellulose (% DM)	37.9 ± 0.33	41.3 ± 0.27
ADL (% DM)	6.81 ± 0.17	6.99 ± 0.23
pH value	6.88 ± 0.076	5.55 ± 0.043
Lactic acid (% DM)	ND	2.48 ± 0.137
Acetic acid (% DM)	ND	3.40 ± 0.211
Butyric acid (% DM)	ND	2.67 ± 0.173
LAB (log_10_ CFU/g FM)	3.52 ± 0.127	6.87 ± 0.393
AB (log_10_ CFU/g FM)	6.86 ± 0.251	6.13 ± 0.174
Molds (log_10_ CFU/g FM)	5.12 ± 0.181	ND

DM, dry matter; WSC, water-soluble carbohydrates; NDS, neutral detergent solute; ADL, acid detergent lignin; LAB, lactic acid bacteria; AB, aerobic bacteria; FM, fresh matter; ND, not detected; mean ± SD (*n* = 3); values with different small letters differed (*p* < 0.05) among treatment.

**Table 2:** Correlation between the chemical composition of wheat straw with different treatments.

Treatment	Items	NDS	Hemicellulose	Cellulose	Lignin
Wheat straw	NDS	1	−0.527	−0.828 **	−0.666 *
	Hemicellulose	−0.527	1	0.061	−0.138
	Cellulose	−0.828 **	0.061	1	0.642 *
	Lignin	−0.666 *	−0.138	0.642 *	1
Ensiled wheat straw	NDS	1	−0.928 **	−0.720 *	−0.918 **
	Hemicellulose	−0.928 **	1	0.422	0.920 **
	Cellulose	−0.720 *	0.422	1	0.467
	Lignin	−0.918 **	0.920 **	0.467	1

NDS, neutral detergent solute; ** *p* < 0.01; * *p* < 0.05.

## Data Availability

Not applicable.
